# Lung abscess secondary to lung cancer with a coinfection of Granulicatellaadiacens and other bacteria: a case report

**DOI:** 10.1186/s12879-021-06191-8

**Published:** 2021-07-08

**Authors:** Shuo Yang, Liangliang Wu, Lili Xu, Xiang Huang, Xiaofeng Sun, Lan Yang, Ling Xu

**Affiliations:** 1 The first Clinical College of Xinjiang Medical University, Urumqi city, China; 2 Gansu University of traditional Chinese Medicine, Lanzhou, Gansu China; 3 Infection center, the First Affiliated Hospital of Xinjiang Medical University, Urumqi city, China

**Keywords:** Granulicatella adiacens, *Eikenella corrodens*, Lung abscess, Lung cancer, Case report

## Abstract

**Background:**

Granulicatella adiacens is facultative anaerobic Gram-positive bacteria, which mainly involve bacterial endocarditis and bacteremia, but there are few reports of local suppurative infection. A case of lung abscess with a coinfection of Granulicatella adiacens and other bacteria in a lung cancer patient will be reported in this paper. To our knowledge, this is the first case report describing lung abscess due to G.adiacens.

**Case presentation:**

A 52-year-old Chinese woman was admitted to the hospital, She complained of coughing and expectoration for 1 month, shortness of breath for half a month, and dyspnea for 1 day. After a series of examinations, she was diagnosed with lung abscess, pleural effusion, and bronchogenic carcinoma. Draining pus culture demonstrated Granulicatella adiacens. After more than 5 weeks of antibiotic therapies in total, she gradually recovered to fight against lung cancer.

**Conclusion:**

This is the first reported lung abscess caused by G.adiacens. In immunosuppressed hosts, G.adiacens is a virulent pathogen associated with a spectrum of intrathoracic suppurative. Earlier diagnosis and proper drainage surgery with effective antibiotics treatment are very important, and antimicrobial treatment should be more than 5 weeks. When complex pulmonary infection interferes with the CT diagnosis, clinical suspicion of lung cancer should be increased if G.adiacens or *Eikenella corrodens* is detected from a pulmonary infection.

## Background

A lung abscess is defined as a circumscribed area of pus in the lung, which leads to cavity formation and a radiographic finding of an air-fluid in the cavity [1, 2]. In the past decades, anaerobic bacteria and Streptococcus have been the main type of bacteria in lung abscesses [3]. Granulicatella adiacens is facultative anaerobic Gram-positive bacteria and typically involved in cases of bacterial endocarditis [4–7] and bacteremia [8, 9]. There are few reports of local suppurative infection, only a few isolated reports of suppurative osteomyelitis, arthritis, encephalitis, ophthalmia, and pulpitis [10–15]. Here, we report a case of lung abscess with a coinfection of G.adiacens and other bacterias in a lung cancer patient. To our knowledge, this is the first case report describing lung abscess due to G.adiacens.

## Case presentation

**Fig. 1:**
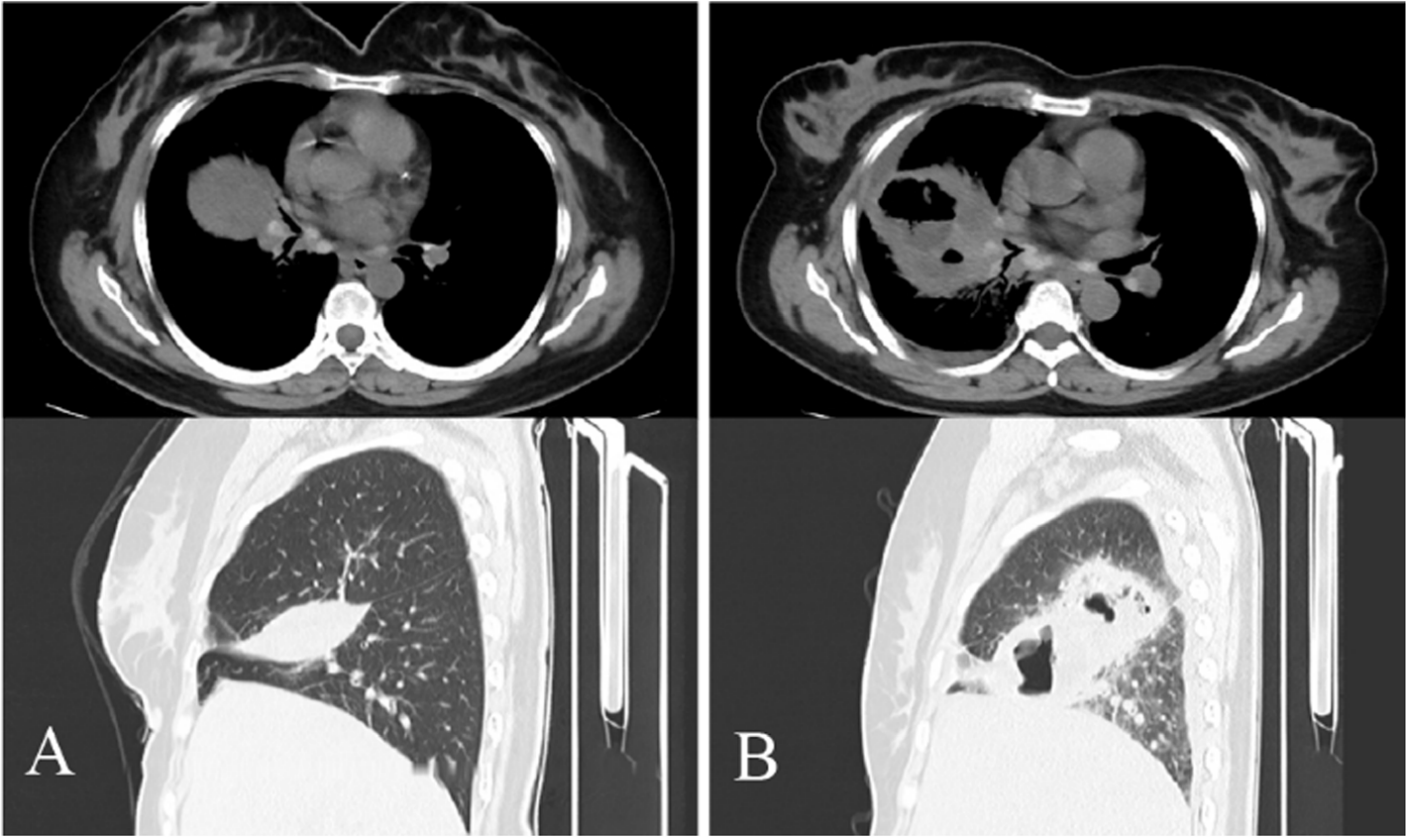
CT showed oblique fissure effusion of right lung on Oct. 8 (**a**) and showed huge thick-walled cavity in the right lung on Oct. 31 (**b**)

On October 8, 2020, a 52-year-old Chinese female was admitted to a local state hospital with cough and expectoration, and a chest CT scan revealed an oblique fissure effusion in the right lung (Fig. 1a), which was diagnosed as a pulmonary infection and treated empirically with levofloxacin at the local state hospital. After 1 week of treatment with this regimen, the patient developed shortness of breath, and the supervising physician adjusted the treatment regimen to levofloxacin combined with latamoxef sodium considering poor infection control. After another 1 week of anti-infective treatment, the patient felt that her symptoms were relieved and asked to be discharged from the hospital and stopped taking antibiotics on her own. After discharge, the patient’s shortness of breath gradually worsened, and she went to the local state hospital again on October 31, and a repeat CT scan showed a large thick-walled cavity in the right lung (Fig. 1b). Outpatient doctors suggested that the patient continue hospitalization. On November 1st, the patient suffered from sudden pain in the right chest and difficulty breathing during hospitalization, so she was transferred to our hospital in the emergency department. She used to have type 2 diabetes without a history of joint pain, heart valvular disease, lung surgery, and no family history of the tumor.

**Fig. 2:**
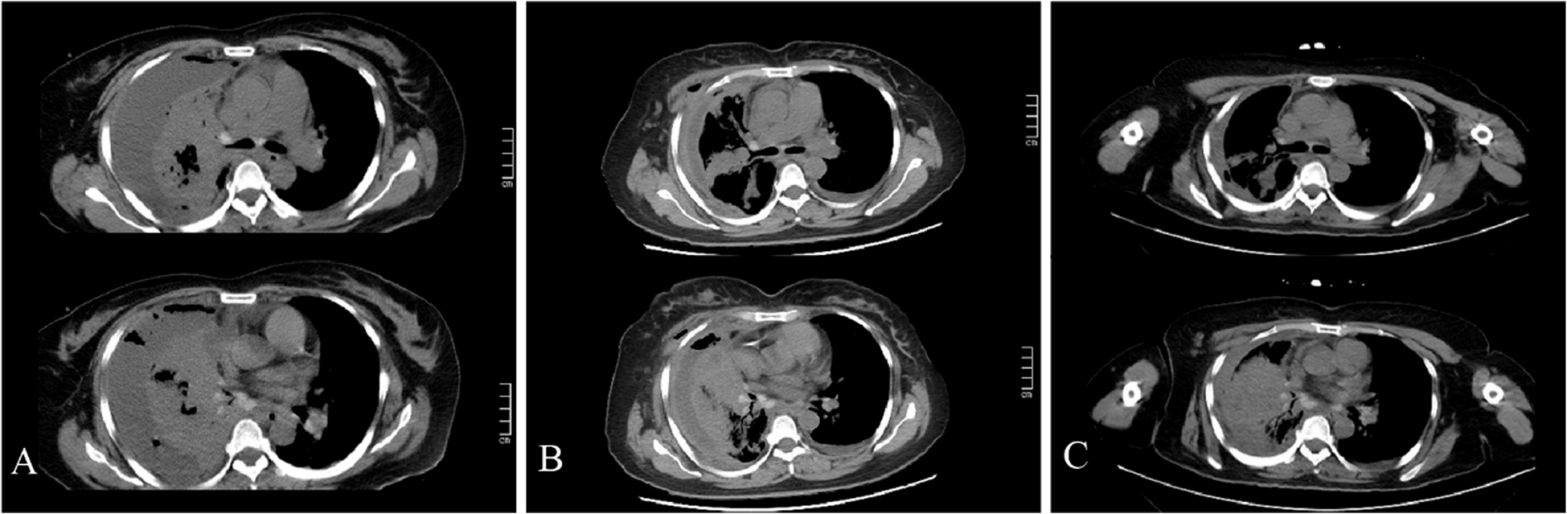
CT showed hydropneumothorax and extensive compression atelectasis on the right side of the chest on November 1st (**a**). After thorough pleural drainage and anti-infective treatment, CT showed right pleural thickening, pleural effusion, multiple cord shadows in the middle and lower lobe, and consolidation of the lower lobe on November 18 (**b**). On December 7, CT re-examination showed that the multiple cords in the middle and lower lobe of the right lung were obviously absorbed and the consolidation range of the lower lobe was slightly reduced

Once admitted, the patient underwent re-examination by CT that revealed right hydropneumothorax and extensive compression atelectasis (Fig. 2a), and then was diagnosed with pyothorax. Moreover, laboratory inspection including WWBC 6.5 × 109/L, PLT 298 × 109/L, IL-6955.2 pg/mL, PCT 0.99 ng/mL, CRP > 90 mg/L, ALB33.02 g/L, indicated infection and malnutrition, and empirical treatment of moxifloxacin were given in the emergency. On November 3, she was admitted to the intensive care unit. The ultrasonic examination showed that the localized anechoic area of 13.0 cm × 10.1 cm × 6.2 cm in the right thoracic cavity and she was given moxifloxacin plus thoracic puncture and drainage. The drainage was porridge-like, with a fishy smell, and the pus was sent for biochemical examination (Table 1). Dyspnea was improved after drainage of 1300 ml pleural effusion for 2 days.

**Table 1:** The biochemistry of pleural effusion suggests empyema, but does not rule out the possibility of tumor

Pleural effusion biochemistry		
Item	outcome	unit
Appearancefaint	yellow	
diaphaneity	turbid	
Rivalta	+	
WBCC	13,531.0	10^6^ /L
Protein	48.20	g/L
LDH	3237.00	U/L
ADA	81.50	U/L

**Fig. 3:**
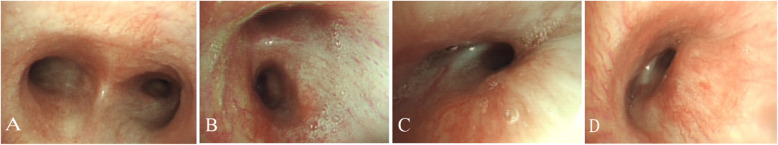
Tracheal Carina (**a**), right common bronchus (**b**), right intermediate bronchus (**c** and **d**); external compressional stenosis of the right intermediate bronchus is seen in the figure

**Fig. 4:**
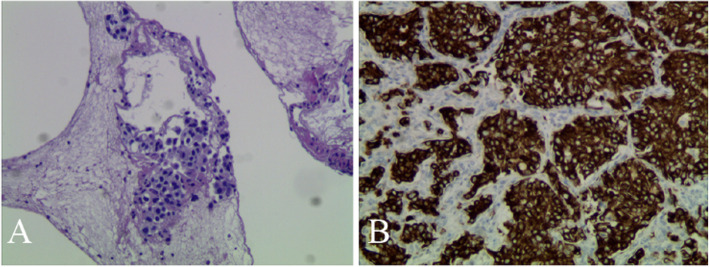
Pathological examination of bronchial tissue in the lower lobe of the right lung shows adenocarcinoma

On November 6, she was transferred to the general ward and continued to be treated with broad-spectrum moxifloxacin. At the same time, fibrinolytic drugs were injected into the pleural cavity for 72 h. During the treatment, the symptoms of shortness of breath worsened again, and ultrasound re-examination showed that the localized anechoic area of 12.5c × 6.8 cm × 6.7 cm was in the right thoracic cavity. The drainage device was replaced by closed thoracic drainage and intermittent drainage of 1000 ml pleural effusion. The drainage fluid was sent to 2 groups of blood culture on different days (each group included an aerobic bottle and an anaerobic bottle). The equipment is a two-way blood culture bottle, consisting of peptone, beef paste, and other culture media, which is suitable for in vitro culture and detection of various aerobic or anaerobic bacteria in body fluids (blood, pleural fluid, etc.). The culture results showed that there were 3 strains of G.adiacens, 1 strain of *Eikenella corrodens*, and 1 strain of *Staphylococcus aureus*. G.adiacens were identified as the main pathogen due to its high detection rate, and the drug sensitivity test showed that it was sensitive to penicillin, erythromycin, vancomycin, cefotaxime, levofloxacin, and linezolid. According to the results of drug sensitivity, we continued to give moxifloxacin treatment (both moxifloxacin and levofloxacin belong to fluoroquinolone antibiotics, with similar antimicrobial spectra and stronger effect of moxifloxacin on cocci and anaerobic bacteria), and additively covered anaerobic bacteria with ornidazole. We also arranged echocardiography and electronic bronchoscopy for the patient. Echocardiography examination showed local calcification of the aortic valve without valvular insufficiency and vegetation, which ruled out endocardial infection. Bronchoscopy examination showed external compressive stenosis of the right middle bronchus (Fig. 3). Besides, minced meat-like tissue of the lower lobe of the right lung was found in the bronchoscopy, and pathological examination revealed adenocarcinoma (Fig. 4). On November 18, CT re-examination showed right pleural thickening, pleural effusion, multiple cord shadows in the middle and lower lobe bottom consolidation (Fig. 2b). After multidisciplinary consultation, it is suggested that surgical intervention should be carried out after the control of pulmonary infection. On November 19, the patient went home with a drainage device and was given oral moxifloxacin and ornidazole as discharge drugs. At the same time, nutritional support lasted throughout the medication period. The patient was revisited On December 7, WBCC and CRP were in the normal range, CT showed right pleural thickening, obvious absorption of multiple cords in the middle and lower lobe of the right lung, and slightly reduced consolidation range of the lower lobe (Fig. 2c). During the telephone follow-up on December 16, we learned that the patient had successfully removed the closed thoracic drainage tube and was receiving further antineoplastic therapy.

## Discussion and conclusions

In the pre-antibiotic era, lung abscess was caused by one type of bacteria, and nowadays almost of 90% cases are caused by multiple bacteria [16, 17]. According to Hammond et al. reported that there is an average of 2.3 different bacteria per abscess [18], while G.adiacens, *Eikenella corrodens*, and *Staphylococcus aureus* were detected in our case. G.adiacens was identified as the main pathogen after 3 times of detection, but the mixed infection of *Eikenella corrodens* and *Staphylococcus aureus* could not be excluded. G.adiacens is a nutritional variant streptococcus (NVS), known as a commensal flora of the human oral, gastrointestinal and genitourinary tracts. It usually grows in chocolate medium or blood medium containing Vit6, L-cysteine, and can also grow around small satellite colonies supported by auxiliary bacteria such as *Staphylococcus aureus* [19]. The microorganism is sometimes excluded by biochemical tests due to its slow growth and generally needs confirmation by molecular techniques [8]. Recently, MALDI-TOF mass spectrometry has been reported as a rapid and exact tool for the identification of G.adiacens [20], and the use of MALDI-TOF mass spectrometry in clinical laboratories has revolutionized routine bacterial identification, making it faster, more accurate, and cheaper [21]. However, the clinical importance of these bacteria is underestimated by clinicians due to the demanding growth and difficult identification of G. advances [22], while the pulmonary infections caused by G.adiacens may be underestimated.

Our patient had no imaging findings related to lung cancer on multiple CT examinations, and if there is no coincidence bronchoscopy, we would almost ignore the fact that she had lung cancer. This could be catastrophic for the patient, because it would cause her to miss the best time to treat cancer. Tumors, malnutrition, and diabetes may be contributing factors of abscess in this case, and tumors may play the most important role. The tumor microenvironment is characterized by increased inhibitory signals and decreased T cells [23], and studies have shown that 62% of lung abscess cases have immunosuppression [24]. Leihao Hu et al. [25] reported an *Eikenella corrodens* infected lung abscess secondary to lung cancer, and coincidentally 3 strain of G.adiacens and 1 strain of *Eikenella corrodens* were detected in our case. Similar to G.adiacens, *Eikenella corrodens* is also a facultative anaerobe colonized in human oral, gastrointestinal and urogenital tract [26]. Immunocompromise due to tumors may allow both species to invade deeper tissues and thrive in carbon dioxide-rich and oxygen-deficient lung tissues. Given the specific habits of these bacteria, clinical suspicion of lung cancer should be increased if G.adiacens or *Eikenella corrodens* are detected in lung infections.

The size of the lung abscess and the location of the right lower lobe are radiological prognostic markers, surgery and catheterization may be considered in patients with a poor prognosis [2], Our patient had a large abscess located mainly in the right lower middle lobe of the lung, which had already invaded the pleural cavity causing massive pleural effusion and extensive compressed pulmonary atelectasis when she came to our hospital, missing the best time for abscess treatment. The American Heart Association (AHA) and the British Society for Antimicrobial Chemotherapy (BSAC) recommend that NVS IE follow the guidelines for enterococcal IE, that is, combined with penicillin or ampicillin plus gentamicin for a duration of 4 to 6 weeks [27, 28]. NVS is increasingly resistant to penicillin, and only 55% of G.adiacens were sensitive to penicillin [29]. Few clinical data support the recommended AHA/BSAC regimen for the treatment of NVS IE [30]. Fanny Quénard et al. showed that antimicrobial treatment (≥8 weeks) is sufficient for prosthetic joint infection caused by G.adiacens [14]. In our patient, after complete chest drainage and up to 5 weeks of moxifloxacin treatment, there was good resorption of inflammation elsewhere in the right lung on CT scan, although solid lesions in the lower and middle segments were poorly resolved, which demonstrates the need for a course of antibiotics beyond 5 weeks in the lung infections caused by G.adiacens.

This is the first reported lung abscess caused by G.adiacens. In immunosuppressed hosts, G.adiacens is a virulent pathogen associated with a spectrum of intrathoracic suppurative. Earlier diagnosis and proper drainage surgery with effective antibiotics treatment are very important, antimicrobial treatment should be more than 5 weeks. When complex pulmonary infection interferes with the CT diagnosis, clinical suspicion of lung cancer should be increased if G.adiacens or *Eikenella corrodens* is detected from a pulmonary infection.

## Data Availability

All the data and materials in this report are from the authors on reasonable request.

## Abbreviations

G.adiacens: Granulicatella adiacens
CT: Computed tomography
WBCC: White blood cell count
PLT: Platelet
IL-6: Interleukin-6
PCT: Procalcitonin
CPR: C-reactive protein
ALB: Albumin
